# A Unique Spectrum of Spontaneous Tumors in *Dino* Knockout Mice Identifies Tissue-Specific Requirements for Tumor Suppression

**DOI:** 10.3390/cells11111818

**Published:** 2022-06-02

**Authors:** Christina B. Marney, Erik S. Anderson, Rachel Baum, Adam M. Schmitt

**Affiliations:** Division of Translational Oncology, Department of Radiation Oncology, Memorial Sloan Kettering Cancer Center, New York, NY 10065, USA; marneyc@mskcc.org (C.B.M.); eanders9@bidmc.harvard.edu (E.S.A.); rachel_baum@berkeley.edu (R.B.)

**Keywords:** Dino, long non-coding RNA, lncRNA, p53, tumor suppression, tumor spectrum

## Abstract

Here, we report that Dino, a lncRNA required for p53 signaling, suppresses spontaneous tumorigenesis in mice. *Dino^−/−^* mice develop significantly more malignant tumors than *Dino^+/+^* littermate controls, consisting predominantly of sarcomas, B cell lymphomas and additional rare tumors. While the prevalence of lymphomas and sarcomas in *Dino^−/−^* mice is similar to that of mice with p53 loss, important distinctions emerged. *p53*-null mice predominantly develop T cell lymphomas; however, no spontaneous T cell lymphoma was observed in *Dino^−/−^* mice. Rather than being a phenocopy of the *p53*-null tumor spectrum, spontaneous tumors in *Dino^−/−^* mice resemble the spectrum of human cancers in which *DINO* is recurrently silenced by methylation in a manner that is mutually exclusive with *TP53* alterations, suggesting that similar tissues in human and mouse require *DINO* for tumor suppression. Consistent with a tissue-specific role for Dino in tumor suppression, loss of *Dino* had no impact on the development of radiation-induced T cell lymphoma and oncogene-driven medulloblastoma, tumors that are accelerated by the loss of p53. Taken together, these data indicate that Dino serves as a potent tumor suppressor molecule specific to a select subset of tissues in mice and humans.

## 1. Introduction

Long non-coding RNAs (lncRNAs) have emerged as important regulators of gene expression and chromatin states in normal cellular differentiation and development, where they can broadly direct transcriptional programs by interacting with proteins, chromatin or messenger RNAs to control cellular behavior [1]. While hundreds of genetic knockout animals exist for protein-coding genes, proportionally few have been generated for lncRNA genes despite their great abundance in the genome [2]. Studies of the lncRNA knockout mice have revealed critical roles in embryonic viability and development, fertility, aging and immune system development, although in several circumstances, the mouse phenotype is only apparent after an exogenous stimulus or perturbation is applied [3]. LncRNA activity has been implicated in multiple human diseases, and a large body of work suggests that lncRNAs contribute important roles in the development of cancer [4,5,6]. While many lncRNAs have been shown with in vitro assays, such as cellular proliferation and survival, to regulate key cellular pathways that define the “hallmarks of cancer”, in vivo evidence of oncogenic or tumor suppressor activity of lncRNAs at the organismal level is limited. 

Preserving genomic integrity is critical for both normal cellular health and for suppression of cancer. p53 is the central guardian of the genome that functions as a tumor-suppressive transcription factor onto which multiple stress pathways converge. For example, DNA damage or oncogenic stress signaling converges onto p53 to elicit cellular phenotypes such as cell cycle arrest, senescence or apoptosis [7]. The stabilization of p53 in stress conditions leads to activation and expression of large numbers of p53-responsive protein-coding and non-coding genes, the profile of which can be modified by p53′s interactions with other proteins, post-translational modification of p53 [8] and the activity of a variety of non-coding RNAs, including microRNAs and lncRNAs [9]. While *TP53* is commonly mutated in many solid tumors in adults [10], many types of cancer including childhood [11] and hematological cancers [12] are frequently *TP53*-wildtype at diagnosis, and thus, cancer must have escaped p53-dependent tumor suppression by other mechanisms. The protein-coding target genes responsible for p53-mediated acute cell fate decisions such as cell cycle arrest, senescence and apoptosis are well characterized and include the canonical p53 targets *p21*, *Puma* and *Noxa*. However, these genes are rarely altered in human cancer and are dispensable for tumor suppression in most model systems [13,14], suggesting that other components of the p53 tumor-suppressive pathway are central to the tumor suppressor phenotype and targeted for alteration in tumorigenesis. 

Multiple groups have identified roles for p53-regulated lncRNAs and microRNAs in mediating the downstream effects of the p53 pathway [15], but rigorous genetic proof of lncRNAs performing essential functions in tumor suppression has largely been unavailable. Dino is an evolutionarily conserved lncRNA that is required for the p53 response to DNA damage in both humans and mice. It directly binds p53 protein promoting p53 stability, enhancing p53 signaling and regulating cell cycle arrest, apoptosis and senescence [16]. We have recently identified Dino as a haplo-insufficient tumor suppressor lncRNA in oncogene driven mouse models of tumorigenesis, using oncogenes *E1A*; *HRasV^12^* to transform mouse embryonic fibroblasts into fibrosarcoma, and the well-characterized *Eµ-myc* model of B cell lymphoma [17]. In both systems, loss of one or two alleles of *Dino* significantly accelerated tumorigenesis, similar to loss of p53 [18,19]. 

In this study, we examined the function of Dino in tumor suppression by assessing the susceptibility of *Dino*-null mice to the formation of spontaneous tumors, revealing a predisposition towards the development of soft tissue and bone sarcoma, B lymphomas and other rare tumors, providing to our knowledge the only rigorous, genetic evidence that loss of lncRNA is sufficient to promote spontaneous tumorigenesis. Furthermore, the spectrum of spontaneous tumors in *Dino^−/−^* demonstrated both similarities and differences from the spectrum of tumors in mice with loss of p53. By assessing the role of Dino in suppressing tumorigenesis in other tissue types with well-characterized requirements for p53-mediated tumor suppression, we provide genetic evidence that Dino functions as a tumor suppressor in a subset of mouse tissues in which p53 is an essential tumor suppressor. Finally, we show that the spectrum of spontaneous tumors in *Dino^−/−^* reflects the spectrum of human cancers in which a genetic relationship is evidenced between *TP53* mutations and *DINO* silencing by hypermethylation, indicating that DINO functions as a p53 pathway tumor suppressor lncRNA in similar tissues in mice and humans.

## 2. Materials and Methods

### 2.1. Animals

C57BL/6 *Dino^−/−^* mice (C57BL/6 *Dino^egfp/egfp^*) were previously described [16]. *Dino^+/+^* (10 female and 9 male) and *Dino^−/−^* (15 female and 13 male) animals in the spontaneous tumor cohort were littermates from *Dino^+/−^* breeding pairs. For acute radiation experiments, wildtype C57BL/6 mice (#027) were purchased from Charles River Laboratories, Wilmington, MA, USA. *SmoM2-eYFP^loxP/loxP^* (#005130) and *Math1-Cre* (#011104) mice were purchased from Jackson Laboratory, Bar Harbor, ME, USA. *p53^−/−^* mice were kindly provided by Scott Lowe. All experiments were performed in accordance with the MSKCC Institutional Animal Care and Use Committee (IACUC) protocol.

### 2.2. Pathology

Moribund mice were humanely euthanized, and recovered tissue was fixed in 10% buffered formalin. The tissue was paraffin-embedded, sectioned and stained with hematoxylin and eosin (H&E). Histopathologic analyses were performed by the American College of Veterinary Pathologists (ACVP) board-certified veterinary anatomic pathologists in the Laboratory of Comparative Pathology at Memorial Sloan Kettering Cancer Center (New York, NY, USA). Lymphoid malignancies were diagnosed using B220 and CD3 immunoreactivity to differentiate B and T cell lymphomas. Leiomyosarcoma was confirmed with immunoreactivity to Vimentin and alpha-SMA. Pheochromocytoma was confirmed via Chromogranin A immunopositivity (data not shown).

### 2.3. Total Body Irradiation

For acute DNA damage response experiments, 3 six-week-old male C57BL/6 mice were irradiated with 5Gy (thymus), and 3 postnatal day 16 male mice were irradiated with 10Gy (cerebellum). In each instance, 3 age-matched males were used as non-irradiated controls. Tissue was harvested 6 h post irradiation after humane euthanasia (CO_2_). For irradiation-induced carcinogenesis, 18 *Dino^+/+^* (8 female and 10 male) and 18 *Dino^−/−^* (13 female and 5 male) mice were irradiated once a week for 4 weeks with 1.8 Gy, starting on postnatal day 28 as previously described [20]. All animals were irradiated using a ^137^Cs source Gammacell-40 Animal Irradiator (Best Theratronics Ltd., Ottawa, ON, Canada).

### 2.4. Survival Analysis

Mice were euthanized when showing evidence of disease including immobility, severe ataxia, rapid breathing, ascites or palpable tumors greater than 1 cm^3^, or when required due to conditions such as rectal prolapse and limb fracture. 

### 2.5. RNA Isolation and Quantitative Reverse Transcription-PCR

RNA was isolated from mouse tissue by Trizol (Ambion (Austin, TX, USA), 15596026) chloroform (Thermo Fisher Scientific (Waltham, MA, USA), C298-500) extraction, followed by isopropanol precipitation with sodium acetate (Ambion, AM9740) and Glycoblue (Thermo Fisher Scientific, AM9515). Precipitated RNA was resuspended in water and treated with Turbo DNase (Thermo Fisher Scientific, AM2238), then further purified through an RNAeasy column as per the manufacturer protocol (Qiagen (Germantown, MD, USA), 74106). Quantitative reverse-transcription was carried out using Power SYBR Green RNA-to-Ct 1-step kit (Applied biosystems (Waltham, MA, USA), 4389986) on an Applied Biosystems QuantStudio 6 Flex Real-Time PCR system. Expression was normalized to Beta-actin, and fold change was calculated using the delta-delta Ct method. *N* = 3 for each condition tested, with significance calculated by T-test and error bars showing standard deviation. Primer sequences:

Dino Forward: gcaatggtgtgcctgactat        Dino Reverse: acttctggcttcccagagc

B-Actin Forward: tcctagcaccatgaagatcaagatc       B-actin Reverse: ctgcttgctgatccacatctg

### 2.6. Statistics

Incidences of tumors in wildtype and *Dino^−/−^* animals were analyzed using a 2 × 2 contingency table and a one-tailed Fisher’s exact test since we predicted that *Dino^−/−^* animals would have increased spontaneous tumors compared to wildtype animals given our prior results in oncogene-induced tumor models [17]. Quantitative RT-PCR was analyzed using an unpaired, two-tailed *t*-test. Significance in Kaplan–Meier survival curves was assessed using the Mantel–Cox Log-Rank test. All analyses were carried out in GraphPad Prism 9, and *p* < 0.05 was considered significant. 

## 3. Results

### 3.1. Spontaneous Tumorigenesis in Dino^−/−^ Mice Reveals a Predisposition for Sarcoma

A colony of 28 *Dino^−/−^* and 19 littermate *Dino^+/+^* mice was followed for 3 years until all animals in the cohort had died (see Table S1 for details). Animals with gross tumors were allowed to continue aging until moribund or the tumor reached 1 cm^3^; therefore, overall survival was not a suitable measure of tumor-free survival in this cohort (Figure S1a). To determine whether *Dino* functions as a suppressor of tumorigenesis in vivo, we focused our analysis on the spectrum of tumors identified after death. 

**Figure 1:**
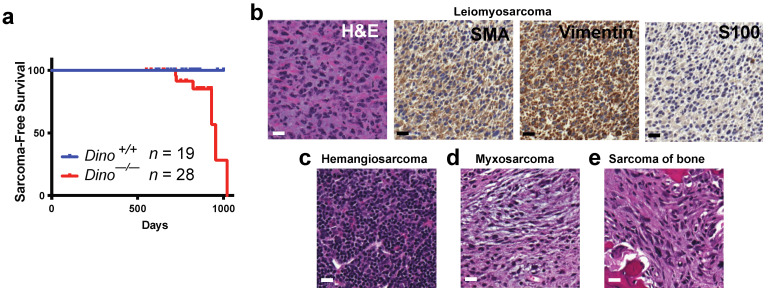
*Dino^−/−^* mice spontaneously develop soft-tissue and bone sarcomas. (**a**) Kaplan–Meier plot of sarcoma free-survival in *Dino^+/+^* and *Dino^−/−^* mice; 6/28 *Dino^−/−^* mice developed sarcoma over their lifespan compared to 0/19 *Dino^+/+^* mice. (**b**) Leiomyosarcoma of the hind limb. Representative images of H&E staining and diagnostic immunohistochemistry showing strong positive staining for vimentin and α-SMA (≥90%) and negative staining for S100 (≤5%). (**c**) Hemangiosarcoma of the liver (H&E). (**d**) Myxosarcoma arising from the muscle of the abdominal wall (H&E). (**e**) Sarcoma of the bone most likely represents osteosarcoma (H&E). All images taken 40X, scale bar = 20 µm.

In total, 78.6% (22/28) of *Dino^−/−^* mice developed a malignant tumor prior to death, significantly more than their *Dino^+/+^* littermates (Table 1, *p* = 0.029). Furthermore, several *Dino^−/−^* mice had multiple distinct cancers. A total of 28 unique malignant tumors were identified in the cohort of 28 mice (an average of 1.0 malignant tumors per mouse). In contrast, 9 mice of 19 in the *Dino^+/+^* littermate cohort developed a total of 10 distinct malignant tumors (an average of 0.53 malignant tumors per mouse). The most frequently observed malignant tumor in both groups was histiocytic sarcoma, a common malignancy of aging C57BL/6 mice [21,22]. Consistent with our previous finding that *Dino* is a haplo-insufficient tumor suppressor in *Eµ-myc* driven B cell lymphoma [17], *Dino^−/−^* mice had nearly a two-fold increased incidence of B cell lymphoma (Figure S1b), although this trend did not reach statistical significance in this small cohort. Moreover, *Dino^−/−^* mice developed a spectrum of spontaneous soft tissue and bone sarcomas not found in littermate animals nor commonly reported in aging C57BL/6 mice [23] (Table 1 (*p* = 0.035), Figure 1a). This included three instances of hemangiosarcoma, one myxosarcoma, one leiomyosarcoma and one sarcoma arising from the bone, most likely representing osteosarcoma (Figure 1b–e). *Dino^−/−^* mice also spontaneously developed one melanoma of the skin and one tubulopapillary adenocarcinoma of the upper gastrointestinal tract, additional tumor types that are rare in the C57BL/6 background (Figure S1c,d). While the loss of p53 has extensively been linked to the accelerated formation of lung adenocarcinoma [24], we observed no increase in the rate of lung adenocarcinoma in *Dino^−/−^* mice. 

These data demonstrate that genetic loss of the lncRNA Dino is sufficient to facilitate tumorigenesis and that *Dino* is a bone fide tumor suppressor capable of preventing the formation of tumors in vivo, the first example of a spontaneous tumor phenotype in a lncRNA knockout mouse to our knowledge. Furthermore, the spectrum of tumors observed in *Dino^−/−^* shared some common features with the spectrum of spontaneous cancers that develop in *p53^+/−^* and *p53^−/−^* mice, with sarcoma and lymphomas being predominant cancer types, consistent with Dino’s role in p53-dependent tumor suppression. 

### 3.2. The Spectrum of Spontaneous Tumors in Dino^−/−^ Mice Is Similar to Types of Human Cancers in Which Epigenetic Silencing of DINO Is Mutually Exclusive with TP53 Mutations

We have previously reported that human *DINO* expression is regulated via methylation in the shore of a CpG island located at the shared *DINO/CDKN1A* locus and that hypermethylation of this region had no effect on the expression of *CDKN1A*. Examining 30 types of human cancers represented in the TCGA dataset, we observed that *TP53* mutations and *DINO* hypermethylation are mutually exclusive cancer alterations in six types of human cancer, including soft tissue sarcoma, skin melanoma, diffuse large B cell lymphoma and stomach adenocarcinoma [17]. Mutual exclusivity of genetic alterations in cancer typically indicates that two genes contribute to the same mechanism and alteration of just one of the genes is sufficient to facilitate tumorigenesis [25], suggesting that loss of *DINO* was sufficient for escape from p53-dependent tumor suppression despite retention of wildtype *TP53*. Intriguingly, we observed that *DINO* hypermethylation is not mutually exclusive with *TP53* mutations in several other types of cancer in which *TP53* is recurrently mutated, including lung adenocarcinoma. That *DINO* hypermethylation is not mutually exclusive with *TP53* mutations in these cancer types suggested that silencing of *DINO* was insufficient to impair the p53-dependent tumor suppressor phenotype in these tissues. 

**Figure 2:**
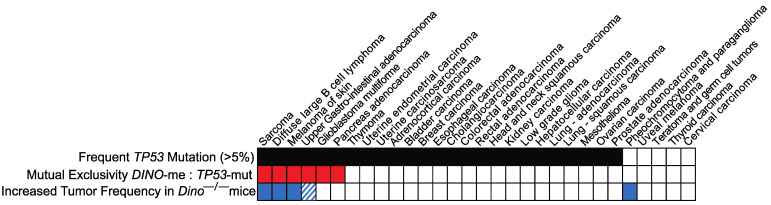
The spectrum of spontaneous tumors in *Dino^−/−^* mice reflects the spectrum of human cancers in which *TP53* mutations and *DINO* hypermethylation are mutually exclusive. Analysis of 30 human tumor types in the TCGA dataset identifies a subset of six tumor types in which *TP53* mutations are mutually exclusive with *DINO* hypermethylation (red). *Dino^−/−^* mice spontaneously developed tumors in 3 of these tumor classifications including sarcoma, diffuse large B cell lymphoma and melanoma of the skin (blue). *DINO* hypermethylation and *TP53* mutations are mutually exclusive in stomach adenocarcinoma, an upper gastrointestinal malignancy, and another upper gastrointestinal adenocarcinoma arising from the jejunum was observed in a *Dino^−/−^* mouse (blue stripes). Fisher’s exact test for an interaction; *p* = 0.001.

The spectrum of spontaneous tumors that developed in *Dino^−/−^* mice was similar to the types of human cancers in which *DINO* silencing is mutually exclusive with *TP53* mutations (Figure 2). We observed spontaneous tumors in *Dino^−/−^* mice corresponding to three of six of the human tumor subtypes found to have mutual exclusivity between *TP53* mutation and *DINO* hypermethylation: sarcoma, B cell lymphoma and skin melanoma (Figure 1b–e and Figure S1b,c). Another rare spontaneous tumor observed in *Dino^−/−^* was an upper gastrointestinal adenocarcinoma arising in the jejunum (Figure S1d). While the TCGA did not examine the genetics of small bowel adenocarcinomas, we did observe that *TP53* mutations and *DINO* hypermethylation were mutually exclusive in stomach adenocarcinoma, another type of adenocarcinoma arising from the upper gastrointestinal tract. The relationship between the spontaneous tumor spectrum of *Dino^−/−^* mice and the human tumor types in which *TP53* mutation and *DINO* hypermethylation are mutually exclusive suggests a similarity in the tissue types in which *DINO* serves as an essential tumor suppressor in humans and in mice. 

### 3.3. Loss of Dino Is Insufficient for Tumorigenesis in Selected Tissues That Depend on p53 for Tumor Suppression

The spectrum of spontaneous tumors in *Dino^−/−^* mice reflects human cancers in which *DINO* hypermethylation and *TP53* mutations are mutually exclusive and was consistent with our prior results that loss of *Dino* enhanced oncogene-induced tumorigenesis in B cell and sarcoma [17]. Therefore, we next set out to examine whether *Dino* functions as an essential tumor suppressor in tissues in which p53 is required, but for which human genetics and spontaneous tumors in *Dino^−/−^* mice did not suggest an essential role for *DINO* in p53-dependent tumor suppression. 

**Figure 3:**
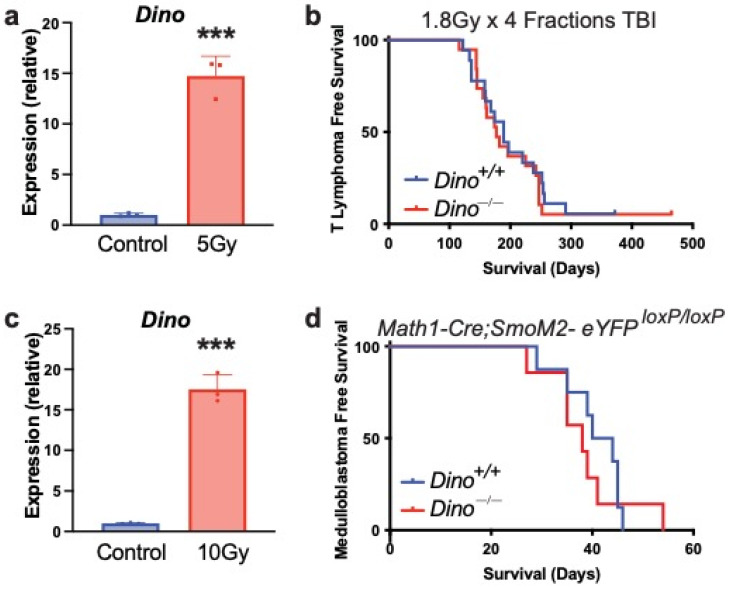
Loss of *Dino* has no effect on tumor-free survival in mouse models of T cell lymphoma or Shh-driven medulloblastoma. (**a**) *Dino* is induced in adult thymus 6 h after 5 Gy TBI. *n* = 3, *** *p* < 0.001, *t*-test. (**b**) Kaplan–Meier curve indicates no significant difference in survival between WT and *Dino* mutant mice post fractionated irradiation. Median thymic lymphoma-free survival *Dino^+/+^* 189 days (*n* = 18), *Dino^−/−^* 177 days (*n* = 18), *p* = 0.6, Log-Rank test. (**c**) *Dino* is induced in postnatal day 16 cerebellum 6 h after 10 Gy irradiation. N = 3, *** *p* < 0.001, *t*-test. (**d**) Kaplan–Meier curve indicates no significant difference in survival between WT and *Dino* mutant mice in a *Math1-Cre; SmoM2- eYFP^loxP/loxP^* background. Median medulloblastoma-free survival *Dino^+/+^* 42 days (*n* = 8), *Dino^−/−^* 38 days (*n* = 7). *p* = 0.62, Log-Rank test.

While *p53*-null mice predominantly develop spontaneous T lymphomas of thymic origin, we were surprised to observe no incidence of T cell lymphoma in *Dino^−/−^* mice (Table 1) [18,26]. Given the well-established role of Dino in p53 pathway regulation in multiple cell types, including T cells [16,17,27], the absence of spontaneous T cell lymphoma in *Dino*-null mice is particularly striking. p53 is critical for the protection of cells against radiation-induced carcinogenesis [28], particularly in the thymus, which undergoes extensive p53-dependent apoptosis after radiation [29,30]. *Dino* is robustly induced in adult mouse thymus after 5Gy TBI (Figure 3a), and we have previously demonstrated that *Dino* is required for radiation-induced apoptosis in thymocytes [16]. The involvement of Dino in p53-dependent cell fate decisions in T cells is expected to be highly suggestive of a tumor-suppressive role for Dino in T cell tumorigenesis. 

To interrogate the role of Dino in suppressing T cell lymphoma directly, we utilized a model of thymic lymphomagenesis induced by exposure to ionizing radiation at a dose that effectively induces thymic T cell lymphomas in *p53^+/+^* mice [31] and which is significantly accelerated by the loss of p53, demonstrating that p53-dependent tumor suppression is essential in this model [28,32]. Consistent with observations in spontaneous tumors, the loss of *Dino* had no effect on T cell lymphoma-free survival after irradiation (Figure 3b). This suggests that although Dino has a functional role in the acute cellular response to radiation in the thymus, it does not function as an essential tumor suppressor in this cell type when constitutively inactivated by genetic deletion.

p53 is also an essential suppressor of tumorigenesis in mouse models of Sonic hedgehog (Shh) medulloblastoma. Loss of p53 significantly accelerates medulloblastoma development in mice with mutations that activate Shh signaling, such as loss of the negative regulator *Patched* [33]. We similarly observed that loss of p53 significantly accelerated medulloblastoma development in mice expressing a constitutively active, oncogenic Smoothened variant, SmoM2c. In this model, mice with a second allele (*Math1-cre*) selectively express SmoM2c in cerebellar granule neurons after recombination excises a LoxP-STOP-LoxP cassette that otherwise prematurely terminates the expression of the SmoM2c allele [34]. Shh medulloblastoma develop at a significantly more rapid rate in *p53^−/−^; Math1-Cre*; *SmoM2-eYFP^loxP/loxP^* mice compared to *p53^+/+^*; *Math1-Cre*; *SmoM2-eYFP^loxP/loxP^* mice (Figure S2), indicating that the p53 pathway is essential to suppression of medulloblastoma formation in this model. We next examined whether Dino is expressed in the developing cerebellum, a region of the brain that is largely composed of cerebellar granule neurons, the cell of origin of Shh medulloblastoma [35,36]. Similar to our observations in thymus and those previously reported for B cells and fibroblasts [17], *Dino* is highly induced by genotoxic stress in the developing cerebellum (Figure 3c). However, genetic loss of *Dino* did not accelerate medulloblastoma development in *Math1-Cre*; *SmoM2-eYFP^loxP/loxP^* mice (Figure 3d), indicating that Dino, unlike p53, does not contribute to an essential tumor suppressor role in cerebellar granule neural precursors. Furthermore, the lack of Dino-mediated tumor suppression in this oncogene-dependent model system underscores that tumor suppression by Dino is independent of the tumor-initiating genotoxic event. Together, these experimental models identify that Dino is dispensable for tumor suppression in T cells and cerebellar granule neural precursors, two tissues in which p53 is an essential tumor suppressor, a stark contrast to observations that Dino is a haplo-insufficient tumor suppressor in B cells and fibroblasts [17].

## 4. Discussion

This study provides evidence that Dino is a bona fide tumor suppressor lncRNA that inhibits cancer formation in a subset of tissues, including mesenchymal cells and B cells, while also being dispensable to the p53-dependent tumor suppressor program in other tissue types, such as T cells and cerebellar granule neural precursors. This study contrasts markedly with most studies characterizing lncRNAs in cancer phenotypes. Currently, most evidence for functional lncRNA activity is suggested by in vitro cellular assays after exogenous over-expression or acute loss-of-function using RNA interference or antisense oligonucleotides rather than through the study of phenotypes arising on an organismal level as a result of gene deletion. Since lncRNA knockout mice often lack overt phenotypes, skepticism has arisen about the relevance of discoveries made in cellular assays to cancer phenotypes in organisms. However, rigorous and carefully designed studies, often utilizing environmental, genotoxic or oncogenic stressors, have revealed important roles for lncRNAs in many contexts. For example, the lncRNA Malat1 has been implicated in both oncogenesis and tumor suppression when examining gene expression profiles in human tumors and in vitro mechanistic studies [37]. However, *Malat1* knockout mice do not appear to have an apparent cancer phenotype [38,39] but instead display defects in the immune response when exposed to multiple pathogens [40,41]. Similarly, genetic deletion of the p53-inducible lncRNA *Neat1* promotes the transformation *of E1A*; *HRas^V12^*-expressing MEFs and enhances the formation of preneoplastic lesions in a *Kras^G12D^*-driven mouse model of pancreatic ductal carcinoma [42]. However, despite ample additional in vitro evidence for the role of Neat1 in tumor initiation and progression in multiple tumor types [43], *Neat1^−/−^* mice are not reported to spontaneously develop tumors [44]. 

The identification of Dino as a suppressor of spontaneous tumor formation in mice definitively establishes Dino as a functional tumor suppressor in vivo, loss of which predisposes mice towards the development of a subset of p53-associated spontaneous tumors. *p53*-null mice predominantly and rapidly develop lymphomas of thymic origin, with a high frequency of sarcomas and B cell lymphomas also reported [18,26]. This characteristic tumor spectrum is recapitulated in multiple p53-pathway associated animals such as *p19^ARF−/−^* [45], *Atm^−/−^* [46] and transgenic *Mdm2*-amplified animals [47]. However, not all canonical p53-pathway genes studied in knockout animals have revealed a spontaneous tumor phenotype. For example, *Cdkn1a/p21* knockout animals are not prone to spontaneous tumor formation when monitored for their entire lifespan, and *p21* loss has been found to be protective in radiation-induced thymic lymphoma [20,32], again emphasizing the distinct role of the Dino lncRNA transcribed from the shared *Dino*/*Cdkn1a* locus in tumor suppression. Deletion of the pro-apoptotic p53-response gene *Puma* is not reported to predispose animals to spontaneous tumor formation but does protect against lesions induced by genotoxic stress [48,49]. It should be noted, however, that *Puma^−/−^* mice were only followed for 6 months, based on the timeline for tumor development in *p53^−/−^* mice. *p53* heterozygous mice have a much longer latency to tumor development and death, developing fewer lymphomas and instead of succumbing to a wider array of sarcomas [18]. A similarly delayed onset of tumor formation and shift in tumor spectrum has been observed in various knock-in *p53* mutant mice strains, including *p53^S23A/S23A^* [50] and *p53^R172P/R172P^* [51]. *Dino^−/−^* mice did not develop early-onset tumors, and yet, they had a significant increase in tumor incidence at necropsy. This raises the possibility that tumor suppressor phenotypes may be missed in mutant strains of both canonical p53-associated protein-coding genes and p53-associated lncRNAs when experimental design assumes a highly penetrant tumor-prone phenotype similar to that of p53 knockout animals and relies on decreased over-all survival as the only indicator of in vivo spontaneous tumor suppression.

The spectrum of spontaneous tumors identified in *Dino^−/−^* mice reveals a predisposition towards sarcoma formation, similar to the spectrum of tumors seen in *p53^−/−^* mice which lack T, B and NK cells [52]. This suggests that *Dino* may be a critical tissue-specific p53-associated tumor suppressor in the development of sarcoma, a finding supported by the mutual exclusivity between *DINO* silencing through DNA methylation and *p53* mutational status in human sarcomas. Indeed, the spectrum of spontaneous tumors developed in *Dino^−/−^* mice closely mirrors the spectrum of human tumors in which *DINO* hypermethylation was mutually exclusive with *TP53* mutations. This further strengthens the argument that the DINO lncRNA is an important modulator of human tumorigenesis and establishes the *Dino^−/−^* mouse as a model that recapitulates observations in human cancers. 

The notable differences in the spontaneous tumor spectrum of *p53^−/−^* and *Dino^−/−^* mice indicate distinct requirements for tumor suppression between tissue types. Nuanced differences in prior reports on the spectrum of spontaneous tumors in mice with different alterations in the p53 protein and p53 tumor suppressor pathway have similarly suggested this, but this has not been thoroughly examined. Work from several groups that have examined the role of acute p53-activation and radiation-induced T cell lymphoma has illustrated the complexity of p53 pathway functions in tumor suppression. In studies using a mouse in which both copies of the *p53* gene had been replaced with one encoding tamoxifen-inducible *p53,* Christophorou et al. concluded that p53 activation at the time of genotoxic treatment with irradiation did not inhibit eventual lymphomagenesis [53]. Similarly, Hinkal et al. demonstrated that inducible deletion of *p53* immediately prior to TBI had no effect on subsequent lymphoma incidence [54]. Both model systems involve a genetic background in which *p53* is either completely absent prior to irradiation or permanently deleted after Cre-induced recombination. Using a compound transgenic mouse in which the *p53* gene remains intact but rapid temporal silencing can be achieved by doxycycline-induced expression of a p53-targeting shRNA, Lee et al. determined that the acute p53 apoptotic response to fractionated irradiation prevents radiation-induced lymphoma in otherwise *p53* wildtype mice [55]. These data contrast markedly with multiple studies in which loss of p53-dependent apoptotic effects significantly accelerates the development of B lymphomas, highlighting the distinct requirements for p53-dependent apoptotic functions in T cells and B cells. That Dino is required for DNA-damage-induced, p53-dependent apoptosis in T cells but loss of *Dino* did not result in spontaneous or radiation-induced T lymphomas may indicate that Dino contributes to p53 pathway functions that are essential for tumor suppression in certain tissues but not others. This conclusion is further supported by the lack of Dino-mediated tumor suppression in Shh-driven medulloblastoma, a tumor in which *p53* inactivation accelerates tumor formation and is associated with the most high-risk human patients [56]. Future studies are needed to understand the mechanism of Dino’s tissue-specific tumor-suppressor phenotype.

## Figures and Tables

**Table 1:** The spectrum of spontaneous malignant tumors in *Dino^+/+^* and *Dino^−/−^* mice.

Tumor	*Dino^+/+^* (%)	*Dino^−/−^* (%)
Histiocytic sarcoma	4 (21.1)	9 (32.1)
Sarcoma (bone and soft tissue)	0 (0)	**6 (21.4) ^1^**
B and non-T cell lymphoma	3 (16.7)	8 (28.6)
T cell lymphoma	0 (0)	0 (0)
Lung adenocarcinoma	2 (10.5)	2 (7.1)
Melanoma	0 (0)	1 (3.6)
Gastrointestinal adenocarcinoma	0 (0)	1 (3.6)
Pheochromocytoma	0 (0)	1 (3.6)
Total Mice with Tumors	9 (47.4)	**22 (78.6) ^2^**

^1^ *p* = 0.035; ^2^ *p* = 0.029; Fisher’s exact test, one sided.

## Data Availability

All data are available in the main text or the Supplementary Materials.

## Supplementary Materials

The following supporting information can be downloaded at: https://www.mdpi.com/article/10.3390/cells11111818/s1, Figure S1: Overall survival and spontaneous tumors of *Dino^−/−^* mice; p53 suppresses medulloblastoma in the *Math1*-*Cre*; *SmoM2* model. Table S1: Detailed histopathology of spontaneous malignant tumors in *Dino^−/−^* and *Dino^+/+^* littermates.
