# Supplementary material for: A Unique Spectrum of Spontaneous Tumors in Dino Knockout Mice Identifies Tissue-Specific Requirements for Tumor Suppression
Source: Cells. 2022 Jun 2;11(11):1818. doi: 10.3390/cells11111818 (PMC9180304; doi:10.3390/cells11111818)
Supplement: Supplementary file 1 [file cells-11-01818-s001.zip › cells-1707116-SI.pdf]

Supplementary Materials

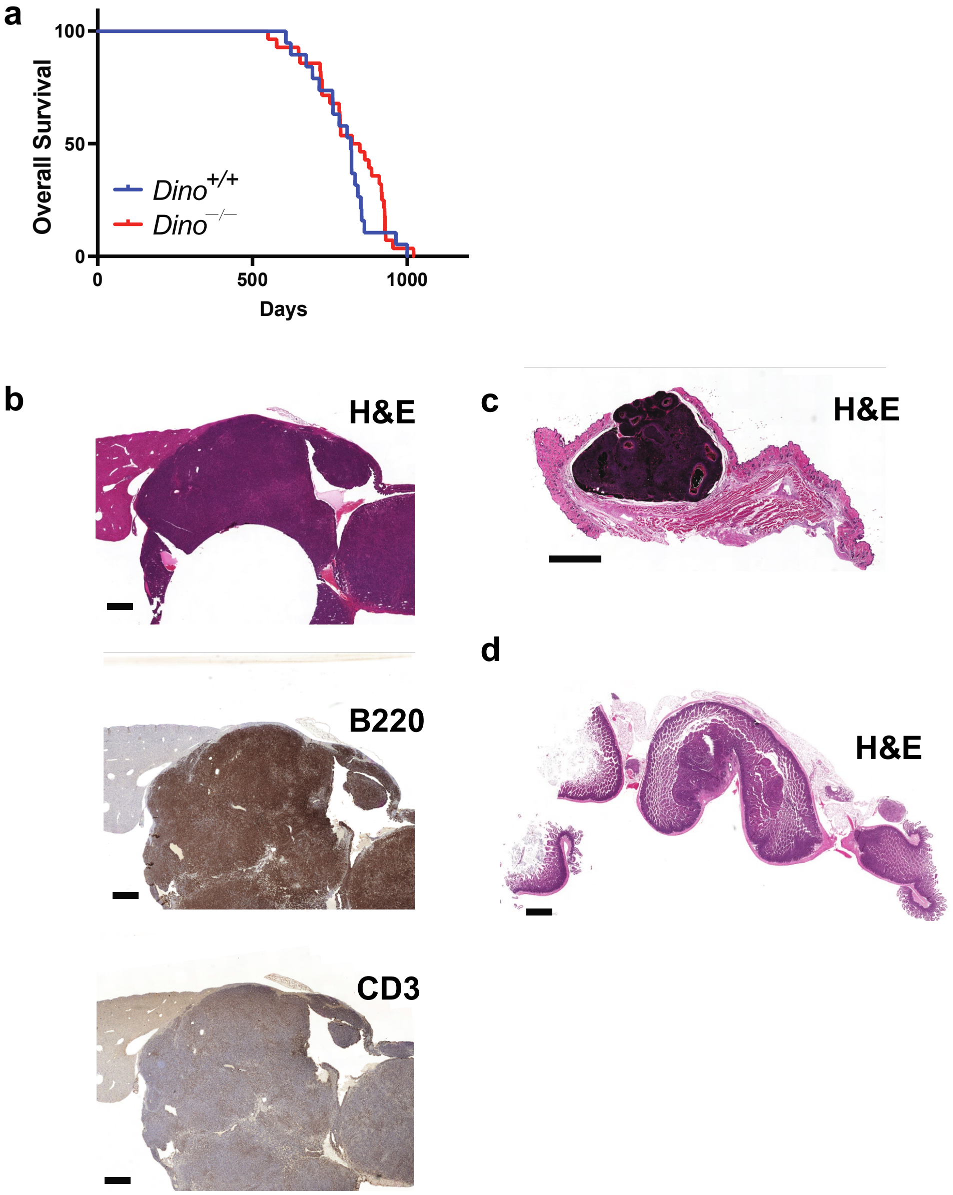

Figure S1: Overall survival and spontaneous tumors of *Dino*$^{-/-}$ mice.

(a) *Dino*$^{-/-}$ mice show no significant difference in overall survival when compared to littermate *Dino*$^{+/+}$ mice. Median survival *Dino*$^{+/+}$ 818 days (*n*=19), *Dino*$^{-/-}$ 835 days (*n*=28), p=0.33, Log-Rank Test.

(b) Representative H&E image of B-cell lymphoma arising in the liver of a *Dino*-null mouse, with corresponding B220 (B cell) and CD3 (T cell) diagnostic IHC used for diagnosis (10X)

(c) Subcutaneous melanoma (H&E, 2X).

(d) Tubulopapillary adenocarcinoma of the jejunum (H&E, 1X).
Scale bar=1000μm

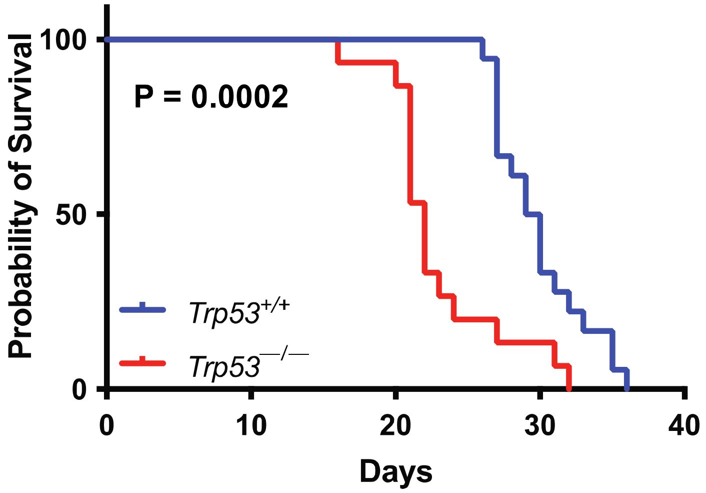

Figure S2: p53 suppresses medulloblastoma in the *Math1*-cre;*SmoM2* model. *p53$^{-/-}$;Math1-Cre;SmoM2-eYFP$^{loxP/loxP}$* mice develop medulloblastoma at a faster rate than *p53$^{+/+}$; Math1-Cre;SmoM2-eYFP$^{loxP/loxP}$* mice. Median survival *p53$^{+/+}$; Math1-Cre;SmoM2-eYFP$^{loxP/loxP}$* 29.5 days (*n*=18), *p53$^{-/-}$; Math1-Cre;SmoM2-eYFP$^{loxP/loxP}$* 22 days (*n*=15), p=0.002, Log-Rank Test.

Table S1: Detailed histopathology of spontaneous malignant tumors in *Dino*$^{-/-}$ and *Dino*$^{+/+}$ littermates.

Genotype	Sex	Age at Death (Days)	Tumor
WT	F	608	Histiocytic Sarcoma
WT	F	674	Bronchioalveolar carcinoma
WT	F	781	Pulmonary Adenocarcinoma
WT	M	759	Diffuse Large B Cell Lymphoma
WT	M	841	Histiocytic Sarcoma, Diffuse Large B Cell Lymphoma
WT	F	820	Histiocytic Sarcoma
WT	F	832	Histiocytic Sarcoma
WT	M	964	Hepatocellular Carcinoma
WT	M	820	B Cell Lymphoma
WT	M	716	
WT	F	624	
WT	F	694	
WT	F	761	
WT	M	818	
WT	F	851	
WT	F	853	
WT	M	806	
WT	M	862	
WT	M	1000	
MUT	M	649	Histiocytic Sarcoma
MUT	M	725	Tail Bone Sarcoma
MUT	M	551	Histiocytic Sarcoma
MUT	F	721	Myxosarcoma
MUT	F	578	Histiocytic Sarcoma, B Cell lymphoma
MUT	F	784	Histiocytic Sarcoma, Pulmonary Adenocarcinoma
MUT	F	782	Atypical Lymphoma (not B or T)
MUT	M	781	Diffuse Large B Cell Lymphoma
MUT	M	785	Histiocytic Sarcoma
MUT	M	847	Diffuse Large B Cell Lymphoma
MUT	M	823	Hemangiosarcoma
MUT	F	910	Histiocytic Sarcoma, B Cell Lymphoma
MUT	F	863	Histiocytic Sarcoma
MUT	M	930	Hemangiosarcoma, Bronchioalveolar Carcinoma, Melanoma
MUT	F	927	Diffuse Large B Cell Lymphoma
MUT	F	876	Diffuse Large B Cell Lymphoma
MUT	F	925	Pheochromocytoma
MUT	F	929	Lymphoma
MUT	F	885	Histiocytic Sarcoma
MUT	F	1021	Leiomyosarcoma
MUT	F	917	Histiocytic Sarcoma, Jejunum Tubulopapillary Adenocarcinoma
MUT	F	954	Hemangiosarcoma
MUT	F	751	
MUT	M	918	
MUT	M	929	
MUT	M	725	
MUT	M	654	
